# Determinants of Breast Cancer Screening Uptake Among Women in Northern Iran: A Cross‐Sectional Analysis of Health Beliefs and Health Literacy

**DOI:** 10.1002/hsr2.72533

**Published:** 2026-05-13

**Authors:** Aida Hosseini, Shabnam Omidvar, Hossein‐Ali Nikbakht, Maryam Nikpour, Farideh Mohsenzadeh‐Ledari

**Affiliations:** ^1^ Student Research Committee Babol University of Medical Sciences Babol Iran; ^2^ Social Determinants of Health Research Center, Health Research Institute Babol University of Medical Sciences Babol Iran; ^3^ Non‐Communicable Pediatric Disease Research Center, Health Research Institute Babol University of Medical Sciences Babol Iran

**Keywords:** breast neoplasms, health belief model, health literacy, mass screening, women

## Abstract

**Background and Aims:**

Breast cancer is the most prevalent malignancy among women worldwide. Early detection through screening is crucial for reducing mortality. This study aimed to determine the association between Health Belief Model (HBM) constructs, health literacy, and breast cancer screening practices among women in Babol, Iran.

**Methods:**

This descriptive‐analytical cross‐sectional study was conducted on 320 women aged 20−65 visiting healthcare centers in Babol in 2024. Data were collected using standardized questionnaires, including demographic information, Champion's Health Belief Model Scale (CHBMS), Breast Fear Scale, and the Health Literacy for Iranian Adults (HELIA) questionnaire. Screening performance was assessed based on breast self‐examination (BSE), clinical breast examination (CBE), and mammography (for women ≥ 40). Data analysis was performed using SPSS version 20, employing descriptive statistics, Chi‐square tests, *t*‐tests, and multivariable logistic regression.

**Results:**

The mean age of participants was 35.73 (± 7.74) years. The rates of BSE (137/320, 42.8%), CBE (124/320, 38.7%), and mammography (in women over 40: 38/104, 36.5%) were observed. Multivariable analysis revealed that performing BSE was significantly associated with higher scores of self‐efficacy (OR = 1.15, 95% CI: 1.10−1.19, *p* < 0.001), positive attitude (OR = 1.10, 95% CI: 1.03−1.16, *p* < 0.001), and cues to action (OR = 1.06, 95% CI: 1.00−1.13, *p* = 0.046), while inversely associated with perceived barriers (OR = 0.96, 95% CI: 0.92−0.99, *p* = 0.048). For CBE, self‐efficacy (OR = 1.15, 95% CI: 1.11−1.20, *p* < 0.001), fear of breast cancer (OR = 0.91, 95% CI: 0.87−0.95, *p* < 0.001), age (OR = 1.11, 95% CI: 1.05−1.17, *p* < 0.001), and attitude (OR = 1.07, 95% CI: 1.01−1.13, *p* = 0.01) were significant predictors. In women over 40, performing mammography was positively associated with self‐efficacy (OR = 1.23, 95% CI: 1.09−1.39, *p* = 0.0012) and age (OR = 1.20, 95% CI: 1.07−1.35, *p* < 0.001).

**Conclusion:**

Breast cancer screening rates were suboptimal. Self‐efficacy was a strong, consistent predictor for all three screening methods (BSE, CBE, and mammography), while other psychological and demographic factors like attitude, fear, and age were uniquely associated with specific screening behaviors.

## 1 Introduction

Responsible for a quarter of total breast cancer incidence in women, it was also the leading diagnosed cancer in women in 2020, and its effect has been rising in much of the world, particularly in transitional countries [1]. About 685,000 women died of breast cancer in 2020, which was 16% or one in every six breast cancer deaths among women [2].

Breast cancer remains the most diagnosed malignancy and a main cause of cancer‐related death in women all over the globe, including Iran [3, 4]. Approximately 14,000 new cases are diagnosed annually in Iran, leading to almost 4000 deaths per annum [5]. While mammography and clinical breast examination (CBE) are recognized screening methods associated with earlier detection of breast cancer, the role of breast self‐examination (BSE) in reducing breast cancer mortality remains uncertain, and it is not currently recommended as an effective standalone early detection strategy [6, 7].

Despite established advantages, attendance at screening programs in Iran is typically suboptimal [8]. There are a number of determinants based on knowledge, attitudes, and socio‐demographic factors influencing screening behaviors. Theoretical frameworks, such as the Health Belief Model (HBM), present a significant starting point to explain these behaviors. Earlier literature on this topic has applied the HBM for examining the factors affecting an individual's participation in breast screening around the world. In a recent systematic review, carried out by Fenech and Gaffiero [9], it was found that perceived barriers were the most powerful predictors of non‐attendance, while cues to action were found to be the facilitators of attending. Perceived benefits were predictors of first‐time attendance and lifetime attendance to breast screening, while “non‐attenders” believed in few benefits, including a lack of belief in early detection through breast screening. Psychological barriers such as fear and anxiety were common among participants [9]. As suggested by the HBM, individuals are more likely to engage in health‐promoting behavior when they perceive themselves as vulnerable to an illness (perceived susceptibility), notice serious potential consequences (perceived severity), believe doing so would have positive effects (perceived benefits), find few or no barriers to doing so (perceived barrier), are encouraged to take action, and possess self‐efficacy in their capacity to engage in the behavior [10, 11]. Despite many national programs, screening attendance among Iranian women with breast cancer is still low. Existing research evidences cultural beliefs, information fallacies, poor perception of danger, and distrust in their capability to carry out BSE or consult mammography as strong deterrents [12, 13, 14, 15].

Although the relationship between HBM constructs and screening behaviors has been studied extensively, results have been heterogeneous. For instance, perceived susceptibility in the context of HBM has been found to be a strong predictor for mammography uptake in some studies [16], whereas others have shown no significant association [17], implying that the degree of predictive power imparted by constructs of HBM may deviate according to culture characteristics, healthcare system profiles, and population risk patterns begins to address. In the same vein, the effect of fear on screening behavior still needs consensus: some studies show evidence supporting fear as a motivator for detecting early changes, while other studies, including our study, identify fear as a significant barrier [18, 19]. The inconsistency between previous research findings demonstrates the need for context‐specific research to examine the complex relationship between psychological, social, and structural factors.

In addition to individual‐level psychological constructs, recent international evidence highlights multilevel influences on screening behavior. Uptake is significantly influenced by health system factors, including access to a primary care provider, availability of screening facilities, costs, and pathways for referrals [20, 21, 22]. Existing literature has indicated that sociocultural norms (e.g., modesty concerns, fatalistic views about cancer, family influence on health decision‐making) among Middle Eastern populations can be particularly salient [19]. Yet few studies explore individual psychological factors, as well as these wider contextual determinants, representing a gap that this study begins to address.

In addition, health literacy (HL)—referring to the way an individual is able to gain, interpret, and understand basic health information required to make informed health decisions—has been identified as an important determinant of health‐related behaviors [21]. Studies have established that inadequate HL impedes screening adherence because it distorts knowledge of screening guidelines, appraisal of risk, and orientation through the healthcare structure [23, 24, 25]. Even though the relationship between components of HBM and screening behaviors has been explored in different contexts [26, 27], and the value of HL has been increasingly validated [28], there has been little research combining these two factors, especially in the Iranian population.

Therefore, the current study was undertaken to evaluate the associations among health beliefs (perceived susceptibility, perceived severity, perceived benefits, perceived barriers, self‐efficacy, and cues to action), fear of breast cancer, HL, and breast examination practices among females who attend healthcare centers in Babol, Iran. The hypothesis of this research is that high levels of HL, high self‐efficacy, high perceived benefits, low perceived barriers, and positive attitude will predict breast screening practice.

These relationships with health beliefs and HL could inform focused strategies to increase screening uptake and, in turn, reduce breast cancer morbidity and mortality.

## 2 Materials and Methods

### 2.1 Study Design and Participants

Five healthcare centers were deliberately chosen from the two municipal districts of Babol to guarantee representation of various socioeconomic backgrounds and patterns of healthcare access. These centers were selected because they cater to catchment areas with differing population densities (urban vs. semi‐urban) and collectively account for approximately 40% of the primary healthcare coverage in the area. Due to logistical limitations and the lack of a comprehensive sampling frame of all eligible women visiting these centers, convenience sampling was utilized, whereby all eligible women who presented during the data collection period were approached in succession until the desired sample size was reached.

#### 2.1.1 Inclusion Criteria

Women aged 20−65 years who were literate (capable of reading and writing in Persian) and had attended the participating healthcare centers during the study time frame.

#### 2.1.2 Exclusion Criteria

(1) A personal history of breast cancer (to prevent potential variations in screening motivations and health beliefs compared to the general population); (2) a personal history of any other malignancy (due to possible confounding effects of cancer‐related screening behaviors); (3) a first‐degree family history of breast cancer (mother, sister, daughter)—these women were excluded as they represent a high‐risk group with different screening recommendations and potentially unique psychological profiles that could skew the evaluation of beliefs and behaviors in the average‐risk population; (4) incomplete questionnaires (defined as more than 20% missing data on any of the primary study instruments)—this criterion is now explicitly outlined in the statistical analysis section as part of the data handling procedures.

Sample size was calculated to be 318 using G*Power software version 3.1.9.7, assuming a logistic regression model with an odds ratio (OR) of 2.11 from a similar study [12], prevalence of screening of 38%, and a statistical power of 95%.

### 2.2 Data Collection Tools

This cross‐sectional study was conducted between January and June 2024. Data was collected using the following instruments:

#### 2.2.1 Demographic and Reproductive Questionnaire

Included age, education, occupation, domicile, marital status, income, age of menarche, and reproductive history.

### 2.3 The Performance Questionnaire of 8 Items

The checklist was created and calibrated by Pour Yusefi and collaborators (CVI = 0.94, CVR = 1).

### 2.4 Operationalization of Screening Outcomes

In alignment with the primary aim of identifying the factors associated with breast cancer screening, we established binary outcome variables for each screening modality based on “ever performed” versus “never performed.” This study targets the primary goal of identifying aspects that are associated with individuals' breast cancer screening uptake. To accompany the measures collected for adherence, we also collected measures of time and frequency during which participants completed their breast cancer screening.

#### 2.4.1 BSE

Participants were asked: “Have you ever performed breast self‐examination?” (yes/no). Individuals who have stated they have performed BSE were then asked: “How often do you perform BSE?” with response options: (1) monthly, (2) every few months, (3) rarely/occasionally. These responses were assessed in two ways: BSE was coded as ever or never performed, and the number of people who perform BSE monthly was computed for the purpose of assessing optimal adherence.

#### 2.4.2 CBE

Participants were asked, “Have you ever had a clinical breast examination by a healthcare provider?” (yes/no). Those answering “yes” were asked: “When was your most recent CBE?” with response options: (1) within the past year, (2) 1−3 years ago, (3) more than 3 years ago. The primary outcome was “ever had CBE” (yes/no).

#### 2.4.3 Mammography

Individuals aged ≥ 40 years were asked: “Have you ever had a mammogram?” (yes/no). Those answering “yes” were asked: “When was your most recent mammogram?” with response options: (1) within the past year, (2) 1−2 years ago, (3) more than 2 years ago. The primary outcome was “ever had mammogram” (yes/no). In the time‐related items, there is one option showing the right performance and the others showing the wrong performance, used to approximate the person's status alone. The reason‐related items are valuable for explaining participation barriers in screening [29].

#### 2.4.4 Champion's Health Belief Model Scale (CHBMS)

The Persian version of the CHBMS, validated by Taymoori et al., was used here [30]. Perceived susceptibility, perceived severity, benefits, barriers, self‐efficacy, and cues to action are measured on a 5‐point Likert scale. Cronbach's *⍺* was 0.81 in the current study.

#### 2.4.5 The Champion Breast Cancer Fear Scale

It is a questionnaire on a 5‐point Likert scale and has eight questions. The range of the total score is from 8 to 40, and a higher score represents a greater fear of breast cancer [31]. In a previous study, the reliability of the scale using Cronbach's *⍺* was also 0.75 [12]. In the present study, Cronbach's *⍺* was calculated to be 0.95.

#### 2.4.6 Health Literacy for Iranian Adults (HELIA) Questionnaire

This 33‐item questionnaire, validated by Montazeri et al., quantifies HL in five domains: reading, access, understanding, appraisal, and decision‐making/behavior [32]. The test score varies from 0 to 100 and is graded as inadequate (0−50), not‐so‐adequate (50.1−66), adequate (66.1−84), and excellent. Cronbach's *⍺* was 0.96.

#### 2.4.7 Knowledge and Attitude Questionnaires

The questionnaires developed by Pouryosofi et al., the knowledge scale comprising 16 questions (example question: “Is it more likely that early detection of breast cancer would cure it? True/False/Don't Know”) and attitude scale with 11 questions (example question: “It is my belief that regular breast cancer screening will help people survive” rated on a 5‐point scale) were used [29]. Cronbach's *⍺* for these scales was 0.83 and 0.74, respectively.

### 2.5 Ethical Considerations

The project was reviewed and approved by the Ethics Committee of Babol University of Medical Sciences (Code: IR.MUBABOL.HRI.REC.1402.124). Written informed consent was also taken from all participants after clarifying the aims of the study and also after ensuring confidentiality.

### 2.6 Statistical Analysis

Prior to analyzing the data set, checks were made for the completeness of the data. Participants with above 20% missing responses on any of the main study instruments (CHBMS, Fear Scale, HELIA, or Knowledge/Attitude questionnaires) were excluded from the analysis (*n* = 12). For the rest of the participants with equal or less than 20% missing data on a given scale, we used mean imputation for that scale, provided the missing items were randomly distributed.

Data distribution was assessed using the Kolmogorov−Smirnov test. All continuous variables were approximately normally distributed; therefore, means and standard deviations are reported throughout. Data analysis was performed using SPSS software version 20 (IBM Corp., Armonk, NY, USA). Descriptive statistics, such as mean, standard deviation, frequency, and percentage, were used. For bivariate analyses, the Chi‐square test, the independent *t*‐test, and the Pearson's correlation coefficient tests were used. All statistical tests were two‐sided. Statistical significance was defined as *α* = 0.05. Variables with *p* < 0.20 in bivariate analyses were considered candidates for multivariable modeling, as recommended by Hosmer and Lemeshow [33].

Model fit was assessed using the Hosmer–Lemeshow test and −2 log‐likelihood values. Multicollinearity among predictors was evaluated, and all variables showed acceptable levels.

OR, along with 95% confidence interval (CI), was reported. A *p* value of less than 0.05 was considered statistically significant.

## 3 Results

The average age of the 320 participants was 35.73 (± 7.74) years. Most participants (67.5%) were less than 40 years of age, 57.5% had high school or lower levels of education, 84.7% currently resided in cities, and 74.7% were married. Most participants (58.1%) had a middle‐level economic condition (Table 1).

**Table 1:** Demographic characteristics of study participants (*n* = 320).

Variable	Frequency (%)	Mean ± SD
Age (years)	—	35.73 ± 7.74
Age group (years)		
20–29	82 (25.6)	
30–39	103 (32.2)	
40–49	85 (26.6)	
≥ 50	50 (15.6)	
Residence		
Urban	218 (68.1)	
Rural	102 (31.9)	
Education		
≤ High school	165 (51.6)	
University/college	155 (48.4)	
Marital status		
Married	274 (85.6)	
Single/divorced/widowed	46 (14.4)	

In the present study, we found the highest and lowest breast cancer screening behavior were related to BSE (42.8%) with (10.3%) adherence to the guidelines and mammography (36.5%) with (29.8%) adherence to the guidelines, respectively (Table 2).

**Table 2:** Breast cancer screening behaviors among participants.

Screening method	Ever performed *n* (%)	Adherent to the guidelines^a^ *N* (%)
Breast self‐examination (BSE)	137 (42.8)	33 (10.3) performed monthly
Clinical breast examination (CBE)	124 (38.7)	73 (22.8) performed annually
Mammography (women ≥ 40 years, *n* = 104)	38 (36.5)	31 (29.8) performed within the past year

^a^ Adherence defined according to Iranian Ministry of Health guidelines: monthly BSE; annual CBE; annual mammography for women ≥ 40 years.

Supporting Information S1: Table 1 presents preliminary correlations between age and screening behaviors. Women aged 40 and older were much more likely to have done a BSE (52.9% vs. 38.0%, *p* = 0.012) and a CBE (61.5% vs. 37.0%, *p* < 0.001). But among those who were screened, following the recommended schedules did not vary by age (*p* = 0.759 for BSE; *p* = 0.852 for CBE). Older women exhibit increased lifetime engagement; however, they do not demonstrate improved routine adherence, underscoring the necessity for interventions to encourage consistent, repeated screening for all age groups.

Table 3 also shows the breast cancer screening behaviors and the predictors among the participants. Self‐efficacy was a highly positive predictor. Specifically, for every one‐point rise on the self‐efficacy score, there was a 15% improvement in the odds of BSE (OR = 1.15, *p* < 0.001). A positive attitude also emerged as being important; a one‐point rise in attitude score resulted in a 10% improvement in the odds of BSE (OR = 1.10, *p* < 0.001). Cues to action had a relatively smaller positive impact, of the order of a one‐point improvement being connected to a 6% improvement in the odds of BSE (OR = 1.06, *p* = 0.046). Residence was also a significant demographic variable: women who lived in rural areas had 2.8 times higher odds of performing BSE compared to women who lived in cities (OR = 2.81, *p* = 0.019).

**Table 3:** Multivariable logistic regression analysis of predictors of breast cancer screening.

	B (SE)	OR (95% CI)	*p* value
Predictors of breast self‐examination			
Self‐efficacy	0.14 (0.02)	1.15 (1.10–1.19)	< 0.001
Positive attitude	0.09 (0.02)	1.10 (1.03–1.16)	< 0.001
Cues to action	0.06 (0.03)	1.06 (1.00–1.13)	0.046
Rural residence	1.03 (0.44)	2.81 (1.18–6.67)	0.019
Predictors of clinical breast examination			
Self‐efficacy	0.14 (0.02)	1.15 (1.11–1.20)	< 0.001
Fear	−0.09 (0.02)	0.91 (0.87–0.95)	< 0.001
Attitude	0.07 (0.02)	1.07 (1.01–1.13)	0.011
Perceived severity	−0.05 (0.02)	0.95 (0.91–0.98)	0.012
Age	0.10 (0.02)	1.11 (1.05–1.17)	< 0.001
Predictors of mammography (≥ 40 years)			
Self‐efficacy	0.06 (0.02)	1.07 (1.01–1.12)	0.012
Age	0.18 (0.05)	1.20 (1.07–1.35)	< 0.001

Abbreviations: B, regression coefficient; Exp(B), odds ratio; SE, standard error.

Self‐efficacy again emerged as a relevant predictor, such that a one‐point increment was associated with a 15% increase in the odds of having a CBE (OR = 1.15, *p* < 0.001). Psychological barriers also stood out as important. A one‐point rise in the score for breast cancer fear was associated with a 9% drop in the odds of undergoing CBE (OR = 0.91, *p* < 0.001). Similarly, a one‐point rise in perceived severity of breast cancer—that is, how serious the woman believed the illness to be—was associated with a 5% reduction in the odds of CBE (OR = 0.95, *p* = 0.012), a counter‐intuitive finding. In contrast, a positive attitude remained a major impetus, increasing the odds of having a CBE by 7% for each one‐point increment (OR = 1.07, *p* = 0.01). Self‐efficacy was the strongest psychological predictor of mammography (women ≥ 40 years). For every one‐point increase in self‐efficacy, the odds of undergoing mammography increased by 23% (OR = 1.23, *p* = 0.001). Age was a significant demographic predictor. For each 1‐year increase in age, the odds of having a mammogram increased by 20% (OR = 1.20, *p* < 0.001) (Table 3).

Utilizing multivariable logistic regression analyses to establish independent predictors of practices of screening, HL did not have a statistically significant relationship with the practice of any of the modalities of screening—BSE, CBE, and mammography (among women aged 40 and above)—upon adjusting for other HBM constructs and sociodemographic variables (*p* > 0.05 for all the models). This means that after adjusting for other sociodemographic and psychological variables, HL did not serve as an independent predictor of adoption of screening.

Self‐efficacy is a consistent and powerful driver across all three screening methods. However, different barriers exist for each: rural residence predicted BSE, while fear was a significant barrier to CBE. For mammography, which is age‐dependent, simply being older was a key predictor of uptake.

Supporting Information S1: Table 2 contrasts HBM constructs, specifically fear, knowledge, attitude, and HL, between women under 40 (*n* = 216) and those aged 40 and above (*n* = 104). Older women exhibited markedly diminished attitude scores (mean difference: 1.55, *p* = 0.023) and not significantly elevated self‐efficacy (MD: −3.49, *p* = 0.129), alongside reduced HL (110.03 vs. 117.35, *p* = 0.380). Perceived susceptibility, severity, barriers, and fear were similar among groups, indicating that although attitudes differ with age, fundamental beliefs regarding breast cancer remain constant.

## 4 Discussion

A primary concern emerging from our data is suboptimal breast cancer screening uptake. Only 42.8% of women made a habit of BSE, and a mere 10.3% of respondents adhered to the monthly recommended regimen. In a similar vein, whilst 38.7% of women had ever had a CBE conducted on them, only one third had it conducted on an annual basis. Most relevantly, for women over the age of 40 for whom mammography is particularly relevant, uptake was substantially below recommended levels at 36.5%. These are well below targets recommended by a multitude of national health authorities and represent a serious public health shortfall. The fact that a majority of participants were well‐educated and of an urban origin—the two factors best correlated with improved healthcare access—means low levels of uptake are particularly concerning. It suggests there are not mere access problems at stake but rather key psychological and knowledge‐related barriers, a hypothesis supported by recognized measures of HL averaging within the “not‐so‐adequate” range.

Overall screening rates were very low, particularly for mammography (36.6%), similar to findings of previous studies conducted in Iran and also in low‐ to middle‐income nations [8, 34, 35, 36]. This reflects a major deficiency in activities for secondary prevention.

The consistent and strong correlation between self‐efficacy and each of the three screening methods supports meta‐analytic research findings that self‐efficacy is one of the strongest predictors of health behaviors among various groups of people [16]. The important and pervasive association of self‐efficacy and each of the three screening methods (BSE, CBE, mammography) is a key outcome of this study. This aligns with the HBM and earlier studies, supporting the notion that a woman's belief in her competence and ability to perform the screening process is a powerful motivator [37, 38]. However, these results differ from recent research in certain western countries which find that perceived barriers and perceived susceptibility are the prevailing predictors [39, 40].

Different cultures may have different expectations for their ability to carry out health‐related behavior, which might account for the differing results between rural and urban women; however, this should be interpreted carefully across all levels of data collected from all subjects. This finding of greater odds of performing BSE for the rural women compared to urban women may represent a compensatory strategy, in that the availability of clinical services was limited (i.e., self‐help in a resource‐limited setting) is also a valid interpretation of the data; however, due to the design of this study it is impossible to ascertain what might be responsible for these differences (e.g., age, formal education, previous history of use of healthcare providers), therefore, the results cannot necessarily be attributed to any one factor. When women have limited options for receiving medical treatment, it is likely that they will turn to self‐treatment methods and that it is therefore very important that those who live in rural areas are given the opportunity to not only receive education on BSE, but also clinical care through CBE and mammograms to which BSE cannot substitute.

Fear has been found to contribute to an individual's avoidance of the CBE versus mammogram screening modality. The significance of fear as either a facilitator [18, 41] of or a barrier [12] to subsequent screening has been demonstrated in previous studies without fully explaining the varying nature of fear across the two modalities. The result of our study regarding the fear being an important barrier is consistent with the systematic review done recently by Fenech and Gaffiero. The important psychological barriers of breast screening internationally included “Fear & Anxiety” (fear of the outcome of screening, pain, procedures, radiation, and embarrassment) [9]. We noted that the experience of fear has a more disruptive effect on access to CBE than it does on access to mammograms (which are technologically advanced and less personal).

The anticipated experience of being physically exposed during a CBE in conservative societies may cause more anxiety than the technological and impersonal aspects of the mammogram experience because of the interpersonal nature of CBE and because of the culturally sensitive nature of CBE and for that reason can magnify fear levels.

Mammography use was best predicted by self‐efficacy and age. The association with age is not unexpected, as older women eligible are closer to the high prevalence age for risk and may interact more readily with the healthcare system. The strong impact of self‐efficacy (7% increase per point) on mammography participation compared to BSE or CBE suggests that as a procedure becomes more complex, intimidating, or technologically advanced, a woman's confidence ever more strongly determines her decision to participate. Even though a significant link between HL and mammography was detected, after adjusting for further variables, this association lost its impact. This finding runs counter to previous studies highlighting the role of HL in the compliance of cancer screening [25, 36, 42, 43]. The effect of HL might be moderated by proximate psychological variables. When variables like self‐efficacy, derived from HL, are included, they account for the variation in mammography uptake, and HL shows no independent impact.

An important finding of this study was the nonsignificance of a noteworthy independent association between HL and breast cancer screening practices in the adjusted models. This is contrary to some previous works, which have stressed the unique role of HL in adhering to cancer screening [44, 45]. A plausible explanation of this discrepancy may be found in the mediating or moderating effect of more proximal psychological factors. The impact of HL on health behaviors may occur indirectly through bolstering key health beliefs such as self‐efficacy, perceived advantages, and reduced perceived barriers. In this study, once those influential mediating variables, self‐efficacy in particular, which was a strong and consistent predictor across all regression equations, had been included in the regression analyses, they likely captured the common variance between HL and screening practices and reduced its independent impact. This speculation is consonant with the logic of the HBM, in which self‐efficacy represents a critical final predictor of behavior. Accordingly, though HL remains an important concept, its impact on screening behaviors in this particular group may be mostly mediated through its impact on selected health beliefs rather than serving directly as an independent predictor. This outcome underscores the importance of designing interventions to simultaneously target both basic HL and specific beliefs, such as self‐efficacy, in order to efficiently improve participation in screening.

### 4.1 Limitations

There are several limitations to this study. Its cross‐sectional design prevented it from ascertaining causal associations between the predictor variables and screening practices. The screen practices were measured on the basis of self‐reported information and so are prone to recall and social desirability biases. The informative but primarily urban and educated sample may not represent broader and less diverse populations. The sub‐group of women eligible for mammography was relatively small (*n* = 104) and should be cautiously interpreted.

Moreover, the study did not include the entire Extended HBM, where health motivation, among other variables, would have enhanced the model's predictive power for the screening behavior. Also, the study's approach emphasized psychological variables at the individual level (e.g., beliefs, fear, attitudes, and HL), yet it did not consider structural obstacles that could affect the screening process. Examples of these obstacles include the availability of transport to screening sites, proximity to screening centers, waiting periods for appointments, costs associated with the screening process, and availability of mammographers of the same gender as the patient. There is evidence that these structural determinants play a vital role in determining screening rates in low‐ and middle‐income countries.

The use of purposive center selection and convenience sampling limits the generalizability of our results. While we made an effort to recruit centers from multiple districts to capture some diversity of centers, the use of non‐probability sampling could introduce bias into our sample. Women who attend healthcare centers may differ from women who do not attend in terms of their health‐seeking behaviors, access to care, and socioeconomic characteristics. As such, the overall rates of screening we found may be an overestimate of screening in comparison to the general population of women in Babol. As a result, our results should be viewed as representative for women who are actively using the primary healthcare system and not for the entire community. Future studies that employ probability‐based sampling methods are necessary to establish these results in a larger population. The exclusion of women with a first‐degree family history of breast cancer, while methodologically appropriate in order to maintain our focus on women at average risk and to prevent confounding by using different recommendations for screening, reduces the generalizability of our results to this important high‐risk group. Women with familial risk may have different health beliefs, fear levels, and screening behaviors that could benefit from a separate study. There is a need for future studies focusing on women with familial risk to be conducted.

## 5 Conclusion

Breast cancer screening among females in Babol is also suboptimal. Self‐efficacy, action cues, attitudes, and HL were significant predictors of BSE, CBE, and mammography. Those interventions augmenting self‐efficacy and HL, particularly among women with low perceived risk for breast cancer, can notably improve take‐up of screening and reduce mortality from breast cancer.

## Author Contributions

**Aida Hosseini:** investigation, data curation, writing – review and editing. **Shabnam Omidvar:** conceptualization, methodology, supervision, writing – original draft, writing – review and editing. **Hossein‐Ali Nikbakht:** software, formal analysis, writing – review and editing. **Maryam Nikpour:** writing – review and editing. **Farideh Mohsenzadeh‐Ledari:** writing – review and editing.

## Funding

This research received no specific grant from any funding agency in the public, commercial, or not‐for‐profit sectors. No funding organization had any role in the study design; collection, analysis, and interpretation of data; writing of the report; or the decision to submit the report for publication.

## Ethics Statement

The project was reviewed and approved by the Ethics Committee of Babol University of Medical Sciences (Code: IR.MUBABOL.HRI.REC.1402.124). Written informed consent was obtained from all participants after clarifying the study's aims and ensuring confidentiality.

## Consent

The authors have nothing to report.

## Conflicts of Interest

The authors declare no conflicts of interest.

## Transparency Statement

The lead author, Shabnam Omidvar, affirms that this manuscript is an honest, accurate, and transparent account of the study being reported; that no important aspects of the study have been omitted; and that any discrepancies from the study as planned (and, if relevant, registered) have been explained.

## Supporting information


**S Table 1:** Association between Breast Cancer Screening Behaviors (BSE and CBE) and Age.
**S Table 2:** Associations between Predictors of Breast Cancer Screenings and Age.

## Data Availability

The data sets used and/or analyzed during the current study are available from the corresponding author upon reasonable request.
