# Supplementary material for: Determinants of Breast Cancer Screening Uptake Among Women in Northern Iran: A Cross‐Sectional Analysis of Health Beliefs and Health Literacy
Source: Health Sci Rep. 2026 May 13;9(5):e72533. doi: 10.1002/hsr2.72533 (PMC13172272; doi:10.1002/hsr2.72533)
Supplement: Supplementary file 1 — S Table 1: Association between Breast Cancer Screening Behaviors (BSE and CBE) and Age. S Table 2: Associations between Predictors of Breast Cancer Screenings and Age. [file HSR2-9-e72533-s001.docx]

-

**Supplementary tables**

**S Table 1: Association between Breast Cancer Screening Behaviors (BSE and CBE) and Age**

| **variables** | | **Age groups** | | **P value** |
| --- | --- | --- | --- | --- |
|  |  | **<40(years)**  **N(%)** | **≥40(Years)**  **N(%)** |  |
| **BSE** | **No** | 134(62.0) | 49(47.1) | 0.012 |
|  | **Yes** | 82(38.0) | 55(52.9) |  |
| **Adherent to the guidelines** | **Performed monthly** | 19(32.2) | 14(25.5) | 0.759 |
|  | **Performed irregular** | 63(76.8) | 41(74.5) |  |
| **CBE** | **No** | 136(63.0) | 40(38.5) | <0.000 |
|  | **Yes** | 80(37.0) | 64(61.5) |  |
| **Adherent to the guidelines** | **Performed annually** | 40(50.0) | 33(51.6) | 0.852 |
|  | **Performed irregularly** | 40(50.0) | 31(48.4) |  |

**S Table 2: Associations between Predictors of Breast Cancer Screenings and Age**

| **Variables** | | **Age Groups** | | **Mean difference (MD) (CI 95%)** | **P value** |
| --- | --- | --- | --- | --- | --- |
|  |  | **<40(years)**  **Mean±sd** | **≥40(Years)**  **Mean±sd** |  |  |
| **Health belief**  **Model** | **Perceived susceptibility** | 22.89±3.12 | 22.28±3.33 | 0.61(-0.14, 1.36) | 0.111 |
|  | **Perceived severity** | 37.97±9.86 | 36.53±11.37 | 1.44(-0.99, 3.88) | 0.245 |
|  | **Cues to action** | 28.10±6.13 | 29.90±5.55 | -1.80(-3.20, -0.40) | 0.667 |
|  | **Perceived benefits** | 34.07±6.77 | 35.67±5.75 | -1.59(-3.11, -0.08) | 0.165 |
|  | **Perceived barriers** | 38.93±12.64 | 40.99±13.27 | -2.06(-5.07, 0.95) | 0.214 |
|  | **Self-efficacy** | 33.22±10.07 | 36.72±11.76 | -3.49(-6.00, -0.99) | 0.129 |
| **Fear** | | 29.10±8.19 | 31.70±7.78 | -2.60(-4.49, -0.71) | 0.334 |
| **Knowledge** | | 35.5±6.10 | 34.88±6.34 | 0.625(-0.82, 2.07) | 0.856 |
| **Attitude** | | 35.48±6.25 | 33.92±7.56 | 1.55(-0.13, 3.24) | 0.023 |
| **Health literacy** | | 117.35±23.55 | 110.03±25.42 | 7.32(1.64, 13.00) | 0.380 |
